# A Pedestrian Detection Scheme Using a Coherent Phase Difference Method Based on 2D Range-Doppler FMCW Radar

**DOI:** 10.3390/s16010124

**Published:** 2016-01-20

**Authors:** Eugin Hyun, Young-Seok Jin, Jong-Hun Lee

**Affiliations:** Advanced Radar Technology (ART) Lab., Division of IoT and Robotics Convergence Research, DGIST, 333 Techno Jungang-daero, Hyeonpung-myeon, Dalseong-gun, Daegu 711-873, Korea; braham@dgist.ac.kr (E.H.); ysjin@dgist.ac.kr (Y.-S.J.)

**Keywords:** FMCW radar, automotive radar, pedestrian detection, 2D FFT, weak target

## Abstract

For an automotive pedestrian detection radar system, fast-ramp based 2D range-Doppler Frequency Modulated Continuous Wave (FMCW) radar is effective for distinguishing between moving targets and unwanted clutter. However, when a weak moving target such as a pedestrian exists together with strong clutter, the pedestrian may be masked by the side-lobe of the clutter even though they are notably separated in the Doppler dimension. To prevent this problem, one popular solution is the use of a windowing scheme with a weighting function. However, this method leads to a spread spectrum, so the pedestrian with weak signal power and slow Doppler may also be masked by the main-lobe of clutter. With a fast-ramp based FMCW radar, if the target is moving, the complex spectrum of the range- Fast Fourier Transform (FFT) is changed with a constant phase difference over ramps. In contrast, the clutter exhibits constant phase irrespective of the ramps. Based on this fact, in this paper we propose a pedestrian detection for highly cluttered environments using a coherent phase difference method. By detecting the coherent phase difference from the complex spectrum of the range-FFT, we first extract the range profile of the moving pedestrians. Then, through the Doppler FFT, we obtain the 2D range-Doppler map for only the pedestrian. To test the proposed detection scheme, we have developed a real-time data logging system with a 24 GHz FMCW transceiver. In laboratory tests, we verified that the signal processing results from the proposed method were much better than those expected from the conventional 2D FFT-based detection method.

## 1. Introduction

Among target detection technologies (*i.e.*, cameras, ultrasonic devices, Lidar), only radar sensors only guarantee system reliability irrespective of environmental conditions (e.g., weather, light, dust). For this reason, radar systems are widely applied in various surveillance applications: defense, automotive, ship, security, and traffic [1]. For the same reasons, radar-based active safety systems have become important key technologies for intelligent automotive systems. These include, for example, forward collision avoidance, blind spot detection, lane-change assistance, rear crash warning, and adaptive cruise control. Recently, there is a demand for radar sensor-based advanced driver assistance systems to provide pedestrian detection to improve traffic safety [2].

For automotive radar systems, Frequency Modulation Continuous Wave (FMCW) radar is generally very popular because the complexity of hardware in the signal processing part can be reduced, compared to that of pulse radar [3,4,5]. In particular, the processing method known as fast-ramp based 2D range-Doppler FMCW Fast Fourier Transform (FFT) is a very effective method.

**Figure 1:**
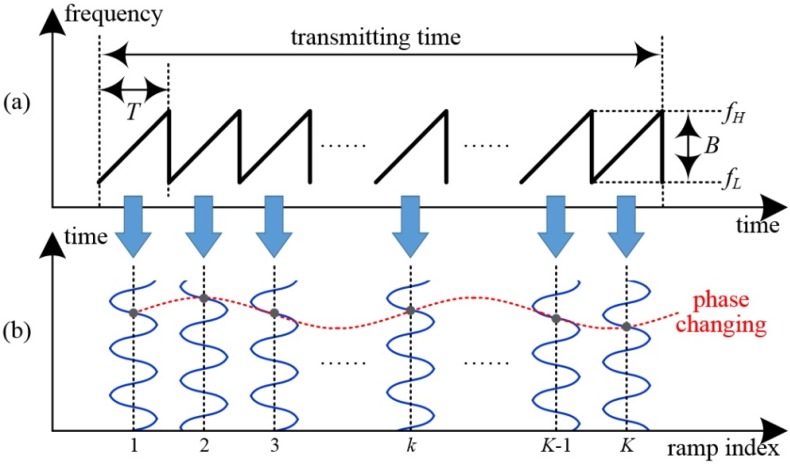
Basic concept of fast-ramp based 2D range-Doppler FMCW radar: (**a**) transmitted signal in the frequency-time domain; and (**b**) beat signal for a single moving target in ramp index domain. Here, T is modulation period, B is bandwidth, $f_{H}$ is maximum instantaneous carrier frequency, $f_{L}$ is the lowest carrier frequency, and K is the number of ramps.

The basic concept of the fast-ramp based FMCW radar is illustrated using the example of a single moving target, as shown in Figure 1. Here, Figure 1a shows the transmitted ramp train, which has a saw-tooth shape in the frequency-time domain. Figure 1b presents the received beat signals reflected from a single moving target in all ramps.

In this method, because the transmission time is very much shorter than the movement time of the target, it can be assumed that the range and radial velocity remain unchangeable in all ramps [6,7,8,9]. Moreover, the reflected Doppler frequency affects the constant phase changing of range-frequency, which is characterized during a single ramp, as shown in Figure 1b. Thus, we can obtain the range-Doppler map by extracting the beat frequencies and phases using 2D FFT processing and distinguish moving targets from clutter [6,7,8,9]. Here, clutter means unwanted echoes with zero Doppler (no motion). However, if the clutter signal is very strong, weak signals from slow-moving targets such as pedestrians could be masked by the side-lobe of the clutter [10].

One aspect of clutter is self-interference generated within the radar transceiver. The typical self-interference is usually caused by antenna mismatch and by insufficient isolation between the transmitting and receiving paths [10]. Another form of internal interference occurs in analogue electronics when the maximum frequency *f*_H_ is switched to the lowest frequency *f*_L_ at the end of each fast ramp (see Figure 1), causing sudden sharp peaks in the signal power [11]. Such forms of self-interference with spectral energy are detected in the near range, spread over neighboring spectral lines, and are superposed on the reflected signals of weakly moving targets such as pedestrians.

Moreover, on real roads, there are many strong echoes from stationary clutter. In that case, the pedestrians may be completely masked by the side-lobe of clutter, even though the clutter and pedestrians have notably separated positions in the Doppler dimension.

To prevent this distortion, one popular solution is to put a weighting function before the FFT [11]. That is, the window provides side-lobe suppression of the spectrum of unwanted echoes in both range and Doppler frequency domains. However, because the window function causes the spectrum to spread over neighboring spectral lines, slow moving targets such as pedestrians may be masked by the broadened main-lobe of the clutter in the Doppler frequency domain. To overcome this problem, it is necessary to provide a method to separate moving pedestrians with weak signal and slow Doppler return from stationary clutter, before using the Doppler FFT.

For this purpose, in this paper, we propose a pedestrian detection scheme for strongly clutter environments, which use a coherent phase difference method in order to suppress stationary echoes, including self-interference. Using the proposed method, it is possible to detect reliably pedestrians who would have previously been masked in clutter.

In Section 2, we propose the coherent phase difference method based pedestrian detection scheme. In Section 3, we present the measurement results tested using a real-time data logging system and 24 GHz FMCW radar transceiver. The conclusions from our study are presented in Section 4.

## 2. Proposed Pedestrian Detection Scheme

**Figure 2:**
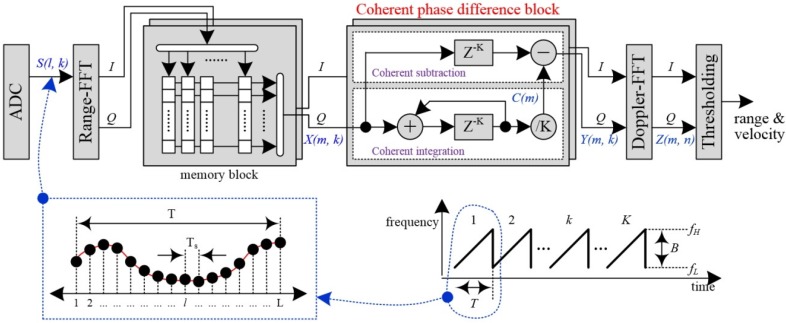
The proposed pedestrian detection scheme using coherent phase difference for 2D range-Doppler FMCW radar.

In this paper, we present our design of the pedestrian detection scheme based on the 2D range-Doppler FMCW radar. The architecture is illustrated in Figure 2.

In the fast-ramp based FMCW radar, the received beat signal is digitalized into {S(l,k), l=1~L, k=1~K} by ADC (Analog Digital Converter) with sampling frequency $f_{s}$. Here, *L* is the sample size in one ramp, and *K* is the number of ramps.

If we assume that a stationary object and a single pedestrian are located in the same range, the received beat signal reflected from two targets is determined by Equation (1), which can be expressed as the summation of the clutter and the moving target [8]:

(1) $$\left(l,k\right)=\left[A_{c}\cdot e^{2\cdot \pi \cdot j\cdot \left\{f_{r}\cdot \left(l-1\right)\right\}}\cdot e^{2\cdot \pi \cdot j\cdot \Delta \varphi_{c}}\right]+\left[A_{m}\cdot e^{2\cdot \pi \cdot j\cdot \left\{f_{r}\cdot \left(l-1\right)\right\}}\cdot e^{2\cdot \pi \cdot j\cdot \left\{f_{D}\cdot T\cdot \left(k-1\right)\right\}}\right]$$

Here, $f_{r}$ is the range-frequency received from two targets. In addition, the Doppler-frequency $f_{D}$ is caused by the radial velocity of pedestrian and depends on angular position of the target away from the line of sight of the radar.

In the first term of Equation (1), $\Delta \varphi_{c}$ is the initial phase of the signal reflected from the clutter (stationary object), and the corresponding amplitude is expressed as $A_{c}$. Here, $\Delta \varphi_{c}$ remains nearly unchangeable over time because the Doppler frequency does not occur.

In contrast, if the target is moving at arbitrary velocity during the transmitting time, the phase of the beat signal received from the moving target changes, as shown by the second term of Equation (1). That is, the coherent phase difference ($\Delta \varphi_{m}=f_{D}\cdot T)$ occurs between two successive ramps, where $A_{m}$ is the reflectivity of the moving target.

The digitalized beat signal S(l,k) is transformed by *M*-point FFT into the frequency domain within each ramp, in order to extract the range spectrum {X(m,k), m=1~M, k=1~K}:

(2) $$X\left(m,k\right)=\overset{L}{\underset{l=1}{\sum }}s\left(l,k\right)\cdot w\left(l\right)\cdot e^{-2\cdot \pi \cdot j\cdot \frac{l}{M}\cdot m}$$

Equation (2) is the mathematical expression for X(m,k), where *L* is the sample size in one ramp, *K* is the number of ramps, and *M* is the index of the range-bins. In addition, the *L*-point window function *w*(l) is also applied before FFT processing. To simplify this description, a rectangular window is used.

In the conventional 2D FFT based approach using windowing, in order to extract a 2D range-Doppler map, the Doppler spectrum {Z(m,n), m=1~M, n=1~N} is estimated by *K*-point windowing and *N*-point FFT processing, as shown in Equation (3):

(3) $$\left(m,n\right)=\overset{K}{\underset{k=1}{\sum }}X\left(m,k\right)\cdot w\left(k\right)\cdot e^{-2\cdot \pi \cdot j\cdot \frac{k}{N}\cdot n}$$

Here, *M* is the index of the range-bins, *K* is the number of ramps, *N* is the index of the Doppler-bins, and *w*(k) is the window function.

If weighting functions are not used for the range-FFT and Doppler-FFT processing of Equations (2) and (3), a weak moving target could be masked by the main-lobe or multiple side-lobes of strong clutter [10]. Thus, windowing is a very useful solution that can be used to suppress the multiple side-lobes of strong clutter in both the range and Doppler frequency domains [11].

However, since the window function causes the spectrum to spread over neighboring spectrum lines, slow moving pedestrians could be masked by the broadened main-lobe of clutter in the Doppler frequency domain.

Therefore, to overcome the problem, in the time domain before Doppler-FFT processing, the components of moving target should be saved and those of the zero Doppler should be suppressed. If this is done, in results of Doppler-FFT, strong clutter can be prevented from occupying the Doppler spectrum and masking any slow moving target.

In this paper, we propose a new pedestrian detection scheme of the 2D range-Doppler FMCW radar and the processing procedure is as follows. First, the proposed coherent phase difference is processed in order to suppress clutter from X(m,k). The proposed block is processed inside a single range-bin over a sequence of adjacent ramps. Here, each sequence of adjacent ramps is selected one by one in a row direction of memory. Thus, the complex output data {Y(m,k), m=1~M, k=1~K} contains only moving target information.

For more detail descriptions, we assume that the range frequency $f_{r}$ occupying two targets correspond to the *r*th range-bin. In this case, we can express the FFT results of the *r*th range-bin as Equation (4):

(4) $$\left(r,k\right)=A_{c}\cdot e^{2\cdot \pi \cdot j\cdot \Delta \varphi_{c}}+A_{m}\cdot e^{2\cdot \pi \cdot j\cdot \Delta \varphi_{m}\cdot \left(k-1\right)}$$

**Figure 3:**
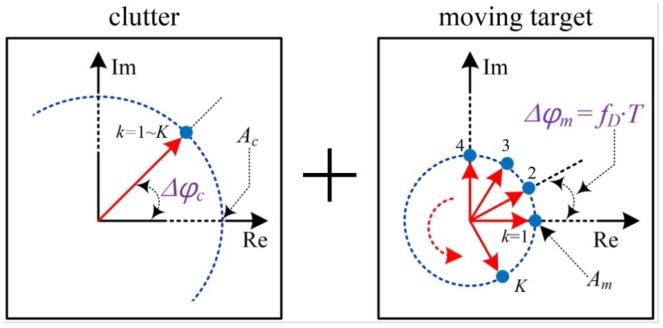
Scheme of the complex results of range-FFT processing in *K* ramps, consisting of clutter and one moving pedestrian at one *r*th range-bin: $f_{D}$ is the detected Doppler-frequency by the radial velocity of the pedestrian, $\Delta \varphi_{c}$ is the constant initial phase of clutter, and $\Delta \varphi_{m}$ is the coherent phase difference of the pedestrian.

Figure 3 illustrates the scheme of the phase shifted samples X(r,1)~X(r,K) in a complex domain. In the Figure 3, the complex results in the *r*th range-bin change with the coherent phase difference $\Delta \varphi_{m}=f_{D}\cdot T$, where the detected Doppler frequency and arbitrary phase difference $\Delta \varphi_{m}$ are proportional to each other. The rotation of the complex vector with increasing phase is caused by the radial velocity of target. In Figure 3, the corresponding magnitude $A_{m}$ is expressed in terms of the size of the circle.

In contrast, the clutter is situated with a constant phase of $\Delta \varphi_{c}$ regardless of the ramp index *K*, as the phases of clutter occupying the same range-bin are unchangeable over the ramps. In Figure 3, the strong magnitude $A_{c}$ of the clutter is also represented as a large circle.

The proposed weak moving detection scheme using a coherent phase difference method is processed in two steps: coherent integration and coherent subtraction. In the first step of coherent integration, we integrate the complex X(r,k) over all ramps, and then the average value is obtained as the accumulated complex values are divided by *K*.

Equation (5) is the mathematical expression for C(r), where C(r) is the complex range profile of unwanted stationary clutter in the *r*th range-bin. In these results, the clutter values may be retained if the magnitude is sufficient, but the phases of the moving target changing over the ramps are cancelled out if *K* is high enough. That is, C(r) is nearly equal to $A_{c}\cdot e^{2\cdot \pi \cdot j\cdot \Delta \varphi_{r}}$:

(5) $$C\left(r\right)=\frac{\sum_{k=1}^{K}X\left(r,k\right)}{K}=A_{c}\cdot e^{2\cdot \pi \cdot j\cdot \Delta \varphi_{c}}+\frac{\sum_{k=1}^{K}A_{m}\cdot e^{2\cdot \pi \cdot j\cdot \Delta \varphi_{m}\cdot \left(k-1\right)}}{K}\approx A_{c}\cdot e^{2\cdot \pi \cdot j\cdot \Delta \varphi_{c}}$$

In the second step of coherent subtraction, according to Equations (4) and (5), we extract the spectrum Y(r,k) of the moving targets by subtracting the range profile C(r) of the unwanted echo from the *r*th complex range profile X(r,k), as expressed by Equation (6). As a result, in the *r*th range-bin, only information about the moving target with coherent phase difference $\Delta \varphi_{m}$ survives in Y(r,k):

(6) $$Y\left(r,k\right)=X\left(r,k\right)-C\left(r\right)\approx A_{m}\cdot e^{j\cdot \Delta \varphi_{m}\cdot \left(k-1\right)}$$

Therefore, by applying all of the procedures into all range-bins, we can complete the process of obtaining Y(m,k), and only moving targets survive with coherent phase differences.

Next, the range spectrum Y(m,k) of moving target is transformed by *K*-point windowing and *N*-point FFT processing for extraction of the Doppler spectrum Z(m,n).

Finally, a thresholding block is carried out to determine whether or not each cell Z(m,n) of 2D range-Doppler is a target. Generally, for adaptive thresholding method, the conventional CA-CFAR (Cell Averaging Constant False Alarm Rate) detector is used in the Doppler dimension [9]. That is, the decision is calculated by averaging the magnitude (Th) of neighboring cells and scaling the value with Equation (7), where α is the scaling factor or the threshold factor:

(7) $$\left|Z\left(m,n\right)\right|=\begin{array}{c} \text{target},\;\text{if}\;\left|Z\left(m,k\right)\right|\geq \alpha \cdot Th\;\\ \text{no}\;\text{target},\;\text{if}\;\left|Z\left(m,k\right)\right|<\alpha \cdot Th \end{array}$$

To investigate the detection performance of the proposed method based on the coherent phase difference, two simulations were conducted in single moving target.

The detection probability and false alarm rate exist in a trade-off relationship depending on the scaling factor of CA-CFAR processing. The scaling factor is also affected by the window size used in the CA-CFAR procedure. Therefore, in this paper, we utilize a scaling factor of 15 as calculated with a window size of 64 and a false alarm rate of 10^−6^, as in reference paper [12]. Moreover, we also select two optional scaling factors of 10 and 20.

In these simulations, we assumed that the pseudo-target was within a range of 1–20 m with a velocity of 4–10 km/h. Each simulation was carried out 1000 times. The used parameters are shown in Table 1 in the next section.

**Figure 4:**
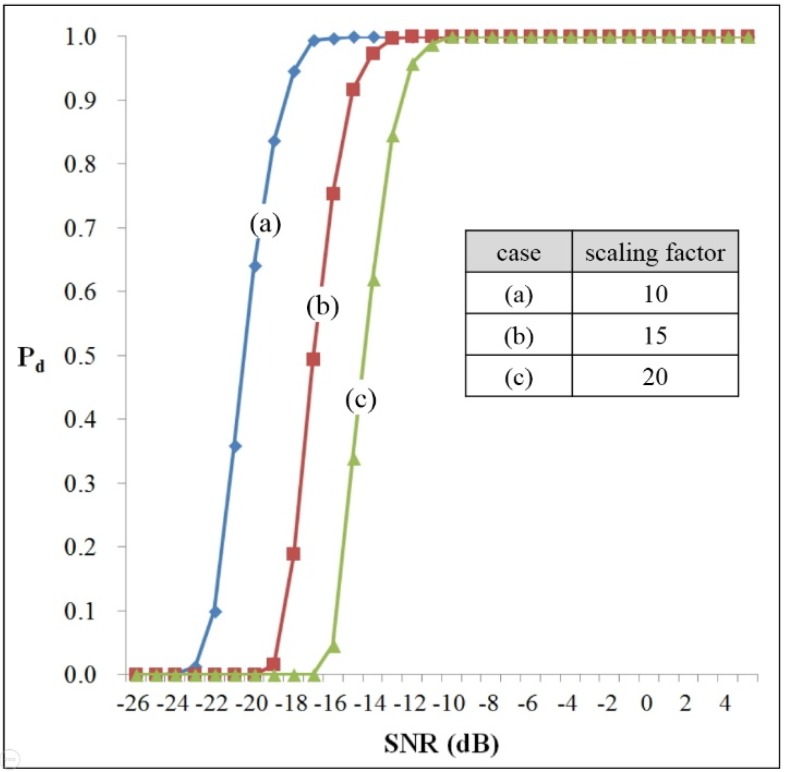
The detection probability of a moving target when varying the scaling factor for the SNR.

First, Figure 4 shows the target detection probability for various Signal-to-Noise Ratios (SNRs) from −26 dB to 5 dB. In this case, we assume that the pseudo-target is moving along middle line of the radar. Here, the *x*-axis indicates the SNR and the *y*-axis is the detection probability (*P_d_*).

In the results, when α is 10 (see case (a)), *P_d_* is dramatically reduced from 90% when the SNR is less −19 dB. It was also noted that the minimum SNR is −14 dB with the proposed method if the required detection probability is more than 95% when the scaling factor is 15 (see case (b)).

**Table 1:** Parameters of the radar system used in this study.

Specification	Symbol	Value
Center frequency (GHz)	*f_c_*	24
Bandwidth (MHz)	*B*	200
Number of transmitting antenna	-	1
Number of receiving antenna	-	1
Modulation period (us)	*T*	80
ADC sampling rate (MHz)	*f_s_*	5
Sample size in one ramp	*L*	200
Number of ramps	*K*	40
Range-FFT point	*M*	512
Doppler-FFT point	*N*	64
Range-bin step size (m)	-	0.59
Velocity-bin step size (km/h)	-	2.2

**Figure 5:**
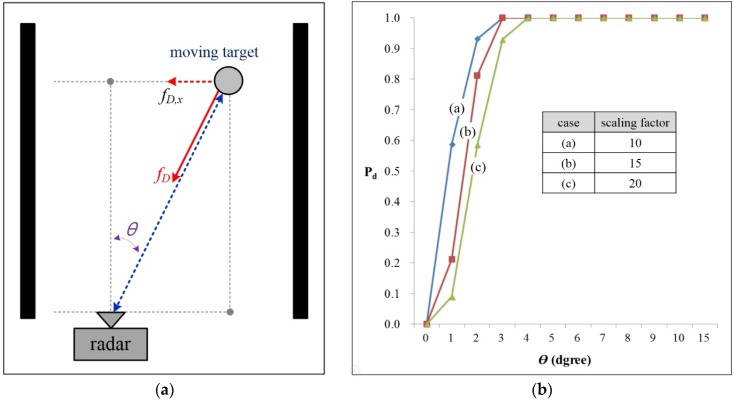
The detection probability of a moving target when varying the scaling factor for the angular position: (**a**) scenario considered for the worst case; and (**b**) simulation results.

Next, we assume that the pedestrian is walking across the azimuth of the radar as in Figure 5a, where θ is the angular position of the target away from the line of sight of the radar, and $f_{D,x}$ are the horizontal Doppler-frequency of the moving target. Thus, Doppler-frequency $f_{D}$ is detected as $f_{D,x}\cdot sin\theta$.

In Figure 5b, the detection performance *versus* the angular position (θ) are presented. Here, the *x*-axis indicates the angular positions (0°–15°) and the *y*-axis is *P_d_*. The simulation is conducted with an SNR of 10 dB. In the results, *P_d_* is reduced as zero when the angular position is less than 2°.

It was noted that the detected velocity of the moving target becomes zero if the moving direction is perpendicular to the line of sight of the radar. Thus, in order to distinguish between clutter and this pedestrian in this worst case, additional algorithms are required for classification and/or tracking. For example, starting from when the instant pedestrian enters the edge of the Field Of View (FOV) to cross the road, angle tracking should be applied so as to indicate this worst case.

In this paper, however, because we only focus on the detection of a moving target as reflected by the coherent phase difference, these additional algorithms for this worst case are out of the scope of this paper.

## 3. Measurement Results

**Figure 6:**
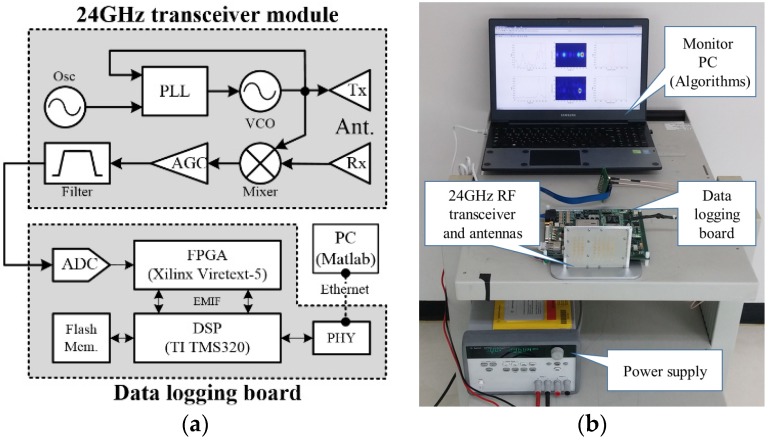
Measurement set-up using real time logging system together with 24 GHz FMCW transceiver: (**a**) block diagram of the developed radar test bed; and (**b**) photo of measurement set-up.

In order to verify the effectiveness of the designed 2D FFT based moving target detection scheme with the proposed coherent phase difference method, we assembled measurement set-up with the newly developed radar system, shown in Figure 6. The measurement set-up included a 24 GHz transceiver module with antennas, a real-time data logging board, and a PC.

We used a 24 GHz transceiver with a single receiving antenna developed for an automotive radar system [13]. The detail system parameters are presented in Table 1.

We designed the specific system parameters including the modulation period to satisfy the 2D range-Doppler FMCW shown in Table 1. That is, since the transmitting time is very much shorter (at 3.2 ms) than the pedestrian movement time, the target movement only affects only the coherent phase difference of each ramp. Moreover, the real-time data logging system was developed based on a hardware board designed in previous work [14].

In this work, we implemented logic by which to synchronize the received data with the modulation period, and to capture the digitalized beat signal in the Field Programmable Gate Array (FPGA). Moreover, instead of direct implementation of the algorithms using a Digtal Signal Processor (DSP) chip, the algorithms were implemented in the PC using Matlab in real time. In future work, we will implement the algorithm into FPGA or DSP.

**Figure 7:**
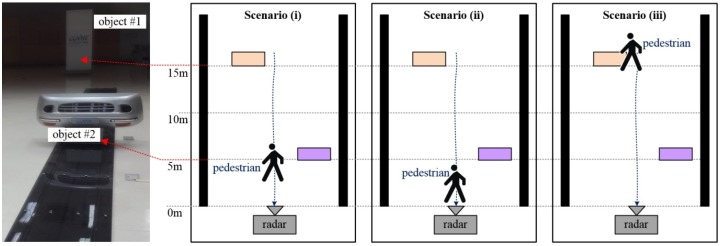
Configuration of the three measurement scenarios for the laboratory test.

We measured the performance of the newly designed pedestrian detection scheme in the laboratory under clutter environments comprising three scenarios. Figure 7 shows the configuration of measurement scenarios for laboratory tests. Two stationary objects with strong signals were located 5 m and 15 m from radar sensor, to provide unwanted clutter. There is a moving human with weak Radar Cross-Section (RCS) within the 1–20 m range.

During the tests, we considered three scenarios:

✓ Scenario (i): Pedestrian walks past the radar at about 3 m distance. In this case, human was not masked by any clutter.
✓ Scenario (ii): Pedestrian walks past the radar at very short range (~1.8 m). Here, the moving human may be masked by self-interference of the radar transceiver.
✓ Scenario (iii): Pedestrian walks past radar at about 15 m distance, next to a stationary object with strong signal. Here, reflections from the nearby stationary object may mask the moving human.

**Figure 8:**
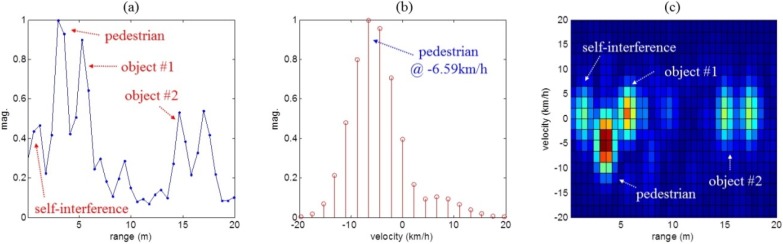

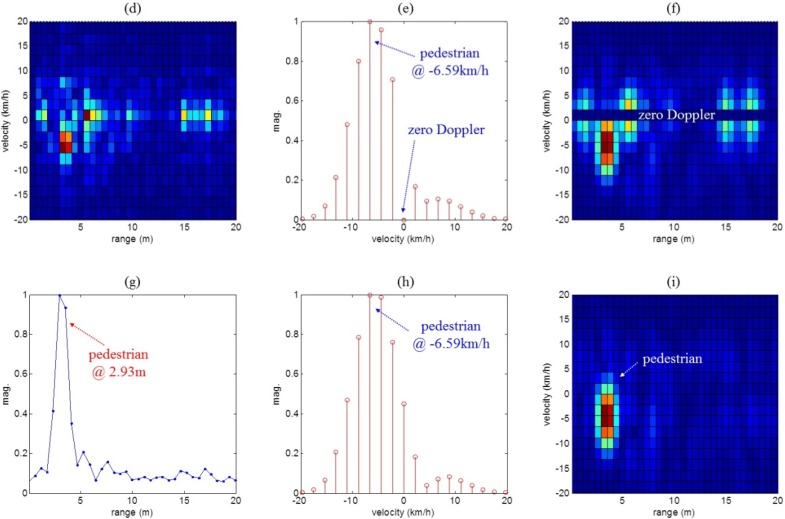
Measurement results obtained using the conventional method and the proposed detection method for scenario (i). Here, (**a**–**c**) present the results of the typical method using windowing; (**d**) indicates the results of the typical method without windowing; (**e**,**f**) show the results of removing the zero Doppler components in the typical method; and (**g**–**i**) are the results of the proposed method.

**Figure 9:**
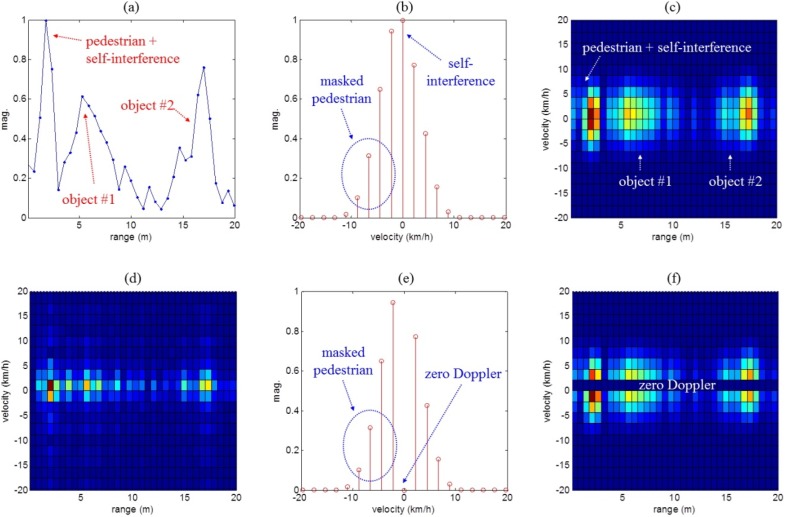

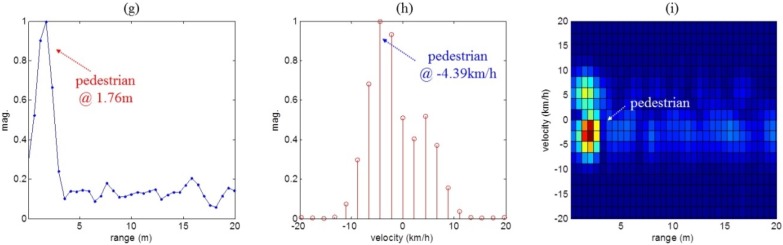
Measurement results using the conventional method and the proposed detection method for scenario (ii). Here, (**a**–**c**) present the results of the typical method using windowing; (**d**) indicates the results of the typical method without windowing; (**e**,**f**) show the results of removing the zero Doppler components in the typical method; and (**g**–**i**) are the results of the proposed method.

**Figure 10:**
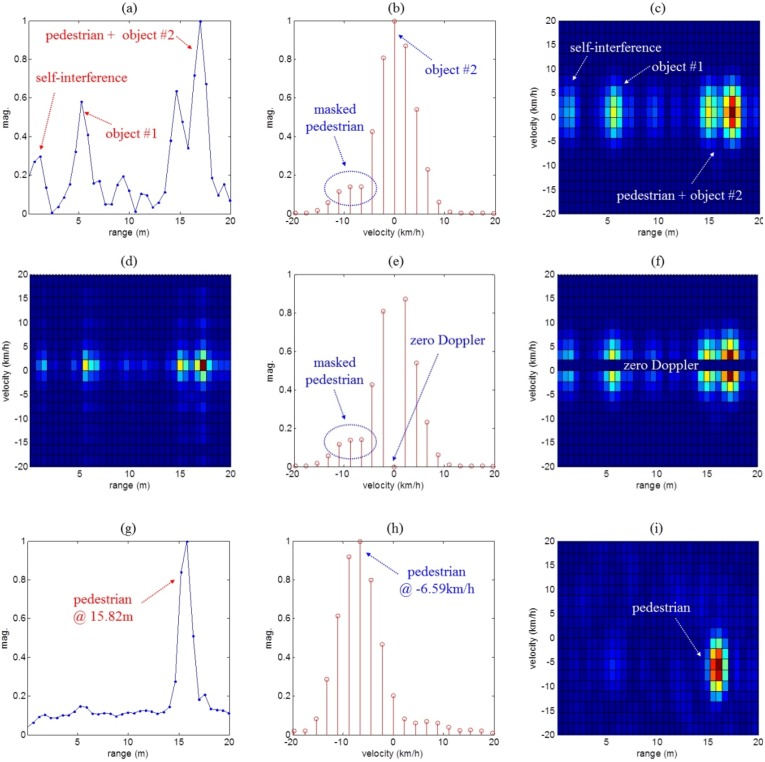
Measurement results using the conventional method and the proposed detection method for scenario (iii). Here, (**a**–**c**) present the results of typical method using windowing; (**d**) indicates the results of the typical method without windowing; (**e**,**f**) show the results of removing the zero Doppler components in the typical method; and (**g**–**i**) are the results of the proposed method.

The results of target detection processing for the three scenarios are shown in Figure 8, Figure 9 and Figure 10, respectively. First, Figure 8a–d, Figure 9a–d and Figure 10a–d show the range and Doppler profiles of the general 2D FFT based target detection algorithm. In these cases, while windowing was applied to obtain the results shown in Figure 8a–c, Figure 9a–c and Figure 10a–c, the range-Doppler maps shown in Figure 8d, Figure 9d and Figure 10d were not obtained using window functions.

In addition, in Figure 8e,f, Figure 9e,f and Figure 10e,f, we removed the zero Doppler components from the results of the typical method with windowing. Next, Figure 8g–i, Figure 9g–i and Figure 10g–i also present results of the proposed method.

In this paper, when applying windowing for range- and Doppler-FFT processing, we employed Hamming and Chebychev windows, respectively.

Figure 8a,g, Figure 9a,g and Figure 10a,g show range profiles, where the *x*-axis indicates range (meter) and the *y*-axis is the normalized magnitude. In Figure 8b,e,h, Figure 9b,e,h and Figure 10b,e,h, we can see Doppler frequency spectra in the specific range occupied by the moving human. Here, the *x*-axis and the *y*-axis indicate velocity (km/h) and normalized magnitude. Finally, 2D range-Doppler maps are presented in Figure 8c,d,f,i, Figure 9c,d,f,i and Figure 10c,d,f,i where the *x*-axis indicates range (meters) and *y*-axis is velocity (km/h).

For the first scenario, Figure 8a shows that the moving human was located in the near range, without masking by self-interference or by reflections from the two stationary objects. Moreover, it was possible to extract the velocity of the human from the Doppler spectrum shown in Figure 8b. Thus, as indicated in Figure 8c, the two sets of clutter and the moving target were placed at different positions of the range and Doppler dimensions.

In order to confirm the windowing effect, we added a range-Doppler map generated without windowing, as shown in Figure 8d. In this result, we can see that many side-lobes were spread out in both the range and Doppler directions; these side-lobes cause a reduction of the target detection performance.

Figure 8e,f show the results of removing the zero Doppler components from the results of the typical method shown in Figure 8b,c. In these cases, we find that the extended main-lobes of clutter remained in the range and Doppler map.

Using the proposed method, two sets of clutter were entirely removed and only information about the moving human remained, as can be seen in the results in Figure 8g–i. In this scenario, because the moving human has as strong enough signal in the near range and is not masked by any of the clutter, both the typical and newly proposed methods can easily separate the moving human from the clutter. Consequentially, compared to the typical methods with or without windowing, we can see that the range-Doppler map of the proposed method is very clear.

For the second experimental scenario, the target range profile of the typical method is presented in Figure 9a. Here, two stationary objects and self-interference appear as strong echoes. Moreover, the location of the slow moving human was masked in the near range by self-interference.

Figure 9b shows the Doppler spectrum at the range (1.76 m) occupied by the moving human. Here, the slow moving human was also masked by the main-lobe of strong clutter (object #1) in the Doppler dimension. In the 2D range-Doppler map shown in Figure 9c, we were not able to determine the range and velocity of the moving target, even though windowing was applied.

Moreover, in Figure 9e,f, even if the zero Doppler components were removed in each range, pedestrian hidden by main-lobes of self-interference could not be easily distinguished in the Doppler frequency domain.

Using the proposed method, we were able to find the moving human at 1.76 m and extract the velocity as −4.39 km/h, as shown in Figure 9g–i. Moreover, strong clutter including self-interference was removed from the range-Doppler map.

Therefore, using the proposed method, we were able to obtain a clearer range-Doppler map than results using two typical methods, in which the window functions are applied or not.

Figure 10 shows the results for the final scenario (iii). In the range profile of Figure 10a is shown that the moving human was masked by strong clutter (from object #2). Especially, in Figure 10b, we could not determine whether the spectra around 7 km/h were produced by strong clutter or by the slow moving human. In the 2D map of Figure 10c, we also could not distinguish the moving human target from the stationary object #2.

As can be seen in Figure 10e,f, due to the spread main-lobe of clutter, it is not easy to separate the pedestrian masked by the object #2, even if we remove the zero Doppler components in the Doppler spectrum.

However, from the results of Figure 10g–i, it can be seen that, using the proposed method, the information about the moving human was easily determined and found to be distance 15.82 m and velocity −6.59 m/s. Therefore, it was proved that range-Doppler map of the proposed method was a reasonable method of pedestrian detection and was superior to the typical methods.

## 4. Conclusions

In this paper, we proposed a pedestrian detection scheme for use in a clutter environment using 2D range-Doppler FMCW radar. A conventional 2D FFT processing radar using fast-ramp based FMCW radar is a very effective method, because it can distinguish a slowly moving target from unwanted clutter.

However, if the clutter has a very strong signal, a moving pedestrian with weak reflectivity and weak Doppler return might be masked by the strong echoes, even though echoes and human target were noticeably separated in the Doppler dimension. Here, we considered self-interference and reflections from stationary targets as clutter.

Thus, we have proposed a method to remove unwanted echoes, to separate slowly moving targets from undesired echoes before Doppler FFT processing. First, to extract the range frequency spectrum of the clutter, the complex values in the same range cells over all ramps are coherently integrated. This is possible because the range-FFT results of moving targets are changed with any coherent phase difference over ramps, but the clutter is located with constant phase irrespective of the ramp. Then, by subtracting the clutter spectrum from the original complex range spectrum, we extract only information about the moving target. Finally, through the Doppler FFT, we obtain the 2D range-Doppler map for the moving targets.

To verify the performance of the proposed algorithms and compare them with the conventional 2D FFT based method, a laboratory tests were conducted for each of three scenarios. For them, we developed the real-time data logging system and 24 GHz FMCW radar transceiver. That is, using the real radar data obtained from measurement system, we verify the target detection algorithms.

The results showed that weak reflections from a slowly moving human were masked by undesired echoes in both range and Doppler dimensions, even though windowing functions were applied to each. Moreover, the results proved that the proposed method effectively extracts information about slowly moving humans from background and internal clutter.

In future, the proposed algorithm could be applied in various automotive safety systems and surveillance systems to support the requirement of multi-target detection. Moreover, additional algorithms should be developed in order to detect pedestrians who move across the azimuth and who are perpendicular to the line of sight of the radar.
